# Risk factors for bronchiolitis hospitalization during the first year of life in a multicenter Italian birth cohort

**DOI:** 10.1186/s13052-015-0149-z

**Published:** 2015-05-26

**Authors:** Marcello Lanari, Federica Prinelli, Fulvio Adorni, Simona Di Santo, Silvia Vandini, Michela Silvestri, Massimo Musicco, and the Study Group of Italian Society of Neonatology on Risk Factors for RSV Hospitalization

**Affiliations:** Pediatrics and Neonatology Unit, Imola Hospital, Imola, Italy; Institute of Biomedical Technologies, National Research Council, Milan, Italy; Department of Food, Environmental and Nutritional Sciences, University of Milan, Milan, Italy; Foundation IRCCS Santa Lucia, Rome, Italy; Neonatology Unit S.Orsola-Malpighi Hospital, Via Massarenti 11, Bologna, Italy; Pediatric Pulmonology and Allergy Unit, Istituto Giannina Gaslini, Genoa, Italy

**Keywords:** Bronchiolitis, Hospitalization, Risk factor, Respiratory syncytial virus, Prophylaxis, Palivizumab, Children

## Abstract

**Background:**

Respiratory Syncytial Virus (RSV) is one of the main causes of respiratory infections during the first year of life. Very premature infants may contract more severe diseases and ‘late preterm infants’ may also be more susceptible to the infection.

The aim of this study is to evaluate the risk factors for hospitalization during the first year of life in children born at different gestational ages in Italy.

**Methods:**

A cohort of 33-34 weeks gestational age (wGA) newborns matched by sex and age with two cohort of newborns born at 35-37 wGA and > 37 wGA were enrolled in this study for a three-year period (2009-2012).

Hospitalization for bronchiolitis (ICD-9 code 466.1) during the first year of life was assessed through phone interview at the end of the RSV season (November–March) and at the completion of the first year of life.

**Results:**

The study enrolled 2314 newborns, of which 2210 (95.5 %) had a one year follow-up and were included in the analysis; 120 (5.4 %) were hospitalized during the first year of life for bronchiolitis. Children born at 33-34 wGA had a higher hospitalization rate compared to the two other groups. The multivariate analysis carried out on the entire population associated the following factors with higher rates for bronchiolitis hospitalization: male gender; prenatal treatment with corticosteroids; prenatal exposure to maternal smoking; singleton delivery; respiratory diseases in neonatal period; surfactant therapy; lack of breastfeeding; siblings <10 years old; living in crowded conditions and/or in unhealthy households and early exposure to the epidemic RSV season. When analysis was restricted to preterms born at 33-34 wGA the following variables were associated to higher rates of bronchiolitis hospitalization: male gender, prenatal exposure to maternal smoking, neonatal surfactant therapy, having siblings <10 years old, living in crowded conditions and being exposed to epidemic season during the first three months of life.

**Conclusion:**

Our study identified some prenatal, perinatal and postnatal conditions proving to be relevant and independent risk factors for hospitalization for bronchiolitis during the first year of life. The combination of these factors may lead to consider palivizumab prophylaxis in Italy.

## Background

Bronchiolitis is the first cause of hospitalization of infants in the USA and it is the most frequent lower respiratory tract disease in preterm infants [1–4].

Infants with chronic lung disease (CLD), congenital heart disease (CHD), immunodeficiency and neuromuscular disorders are particularly at high risk of hospitalization for bronchiolitis [5]. Moreover, preterm infants [6] are also more prone to these infections due to the impaired development of the lung and of the immune system. In particular, it has been observed that prematurity has been associated to an increased likelihood of hospitalization for RSV bronchiolitis [1].

Studies performed in Europe and in the USA have also shown that infants born between 33 and 35 wGA have a higher risk of hospitalization due to RSV infections than full-term infants [1, 7, 8]. This population is at risk of developing severe RSV infection that can result in morbidity and yield similar expenditures to infants born before 33 wGA [1, 9, 10]. Several studies have found other determinants of bronchiolitis and in particular of severe diseases caused by RSV [11], however, two of the largest studies carried out on cohort of infants born at these gestational ages [12–14] reported inconclusive results, probably due to the methodologic approach and differences in context (i.e.social, epidemic, climatic) [8].

In Italy, a multicenter study enrolling over 1200 children younger than two years hospitalized for lower respiratory tract infections (LRTI) found that two-thirds of hospitalizations occurred in children with low birth weight and low gestational age, with an incidence 8.5 % in infants born before 36 weeks vs. 4 % in the general population [15]. Moreover, among children with bronchiolitis, RSV infection was particularly frequent in those born at lower gestational age. Among the patients who were RSV-positive, 60.0 % were born at a gestational age of 33 weeks; 59.1 % between 34–35 wGA; and 47.4 % at more than 35 wGA [15]. This study found that passive tobacco smoke exposure increased the rate of hospitalization for RSV infections (RR 1.4). Moreover, RSV positivity was related to birth order, since it was higher in infants with a larger number of siblings [15].

The aim of the current study is to evaluate the role of prenatal, perinatal and postnatal conditions in determining the risk of hospitalization for bronchiolitis in a large cohort of preterm with GA 33 weeks or more and full term newborns.

In addition to that, the analysis of risk factors was focused on preterm infants born at 33-34 wGA. This population of preterm infants, although well documented to be at high risk of severe RSV infections [1, 7, 8] has been recently crossed out from the revised AAP guidelines [16] for prophylaxis with palivizumab, a monoclonal humanized antibody neutralizing RSV. Two important studies [12–14] analyzed the risk factors for hospitalization in these preterm infants; however, the analysis of risk factors in a large Italian birth cohort has not yet been performed. The determination of national risk factors could improve the capacity to detect newborns at particularly high risk for severe respiratory infection and hospitalization that may be considered for a “tailored” prophylaxis treatment. In Italy prophylaxis with palivizumab is regulated by the Italian Neonatology Society guidelines [17], but they are widely disregarded especially in preterm infants born at 33 wGA or more. The Italian Neonatology Society is reviewing these recommendations through the update of the epidemiological data.

The results of the present multicenter study and the analysis of the risk factors will be considered by the Italian Neonatology Society for the update of the guidelines.

## Methods

This is a multicenter cohort study including consecutive newborns born between 2009 and 2012. All subjects were enrolled at birth and followed up for the first year of life.

Thirty Neonatology Units from hospitals with 1000 or more deliveries per year located in the northern, central, southern and insular regions of Italy participated in this study. Ethic Committees of each participating hospital approved the study protocol. A written consent was obtained from legal guardians of all patients.

For each enrolled newborn of 33-34 wGA, two further newborns of the same sex and with the nearest date of birth were enrolled: one of 35-37 wGA and one of >37 wGA.

Exclusion criteria were life expectancy shorter than six months; haemodynamically significant congenital heart diseases and chronic lung diseases (defined as oxygen requiring at 28 days of life [18]); concurrent enrolment in another trial; programmed or administered RSV prophylaxis with palivizumab and inability to follow up at our center during the study period.

In each center, the enrolling physician filled out a record with prenatal and perinatal data after checking inclusion and exclusion criteria, within two days after the births.

In order to detect possible occurrence of respiratory infections (leading to hospitalization or not), after enrolment a structured phone interview was carried out twice with the parents by a trained interviewer from the Italian National Research Council (CNR). The first interview was carried out at the end of the epidemic RSV season (at end of March in Italy [19]) and the second one at 12 months of age. During the phone interview, postnatal data on potential risk factors such as lack of breastfeeding, exposure to environmental pollution and/or to passive cigarette smoke were recorded.

When parents reported hospitalization of the child for any cause, the enrolling physician collected all the relevant clinical and therapeutic information from the hospital clinical record forms.

The primary outcome was the occurrence of hospitalization/death for bronchiolitis during the first year of life. Hospitalizations for bronchiolitis were defined according to the hospital discharge form with the ICD-9 codes 466.1 (acute bronchiolitis).

Table 1 reports the variables registered by the enrolling physicians and from the interviews concerning neonatal, perinatal and postnatal variables potentially associated to bronchiolitis.

**Table 1:** Baseline demographic and clinical characteristic of the enrolled newborns and of their parents: prenatal conditions

	wGA groups			
	33^+0d^-34^+6d^ (No. 737)	35^+0d^-37^+6d^ (No. 767)	≥38^+0d^ (No. 706)	Total (No. 2210)
Pregnancy				
Assisted reproductive technique	154 (20.9)	90 (11.7)	7 (1.0)	251 (11.4)
Intrauterine growth restriction (IUGR)	82 (11.1)	58 (7.6)	7 (1.0)	147 (6.7)
Mother’s characteristics				
Age >40 year	95 (12.9)	91 (11.9)	46 (6.5)	232 (10.5)
Low education	185 (25.1)	184 (24)	131 (18.6)	500 (22.6)
Nationality				
Italian	654 (88.7)	703 (91.7)	655 (92.8)	2012 (91.0)
African	12 (1.6)	9 (1.2)	8 (1.1)	29 (1.3)
Asian	10 (1.4)	2 (0.3)	6 (0.8)	18 (0.8)
Eastern Europe	36 (4.9)	37 (4.8)	22 (3.1)	95 (4.3)
Western Europe	13 (1.8)	4 (0.5)	7 (1.0)	24 (1.1)
Southern America	12 (1.6)	12 (1.6)	8 (1.1)	32 (1.4)
Maternal diseases during pregnancy	275 (37.3)	220 (28.7)	92 (13.0)	587 (26.6)
Treatment with corticosteroids	404 (54.8)	157 (20.5)	4 (0.6)	565 (25.6)
History of respiratory diseases	138 (18.7)	134 (17.5)	132 (18.7)	404 (18.3)
Asthma	49 (6.6)	50 (6.5)	47 (6.7)	146 (6.6)
COPD	12 (1.6)	8 (1.0)	6 (0.8)	26 (1.2)
Eczema	33 (4.5)	28 (3.7)	26 (3.7)	87 (3.9)
Rhinitis	78 (10.6)	82 (10.7)	90 (12.7)	250 (11.3)
Wheezing	1 (0.1)	-	1 (0.1)	2 (0.1)
Prenatal smoke exposure				
None	559 (75.8)	560 (73.0)	525 (74.4)	1644 (74.4)
To maternal active smoking only	76 (10.3)	93 (12.1)	61 (8.6)	230 (10.4)
To maternal passive smoking only	102 (13.8)	114 (14.9)	120 (17.0)	336 (15.2)
Father’s characteristics				
Age >40 year	184 (25.0)	203 (26.5)	158 (22.4)	545 (24.7)
Low education	219 (29.7)	261 (34.0)	198 (28.0)	678 (30.7)
History of respiratory diseases	138 (18.7)	158 (20.6)	141 (20.0)	437 (19.8)
Asthma	51 (6.9)	48 (6.3)	48 (6.8)	147 (6.7)
COPD	8 (1.1)	14 (1.8)	8 (1.1)	30 (1.4)
Eczema	15 (2.0)	12 (1.6)	15 (2.1)	42 (1.9)
Rhinitis	100 (13.6)	133 (17.3)	117 (16.6)	350 (15.8)

All data are reported as No. (%).wGA = weeks of gestational age; yrs = years

Low level of parental education was defined as no education or primary education (vs. secondary or higher).

Presence of maternal diseases during pregnancy was identified when the mother suffered from chronic conditions or other disorders affecting the pregnancy outcome such as hypertension, preeclampsia or diabetes mellitus.

The presence of parental asthma, COPD and wheezing was recorded when the parents reported to have previously received the diagnosis by other physicians.

A baby was considered small for gestational age (SGA) if smaller than normal for the gestational age, most commonly defined as a weight below the 10^th^ percentile for gestational age, according to the Italian neonatal anthropometric charts [20].

The presence of siblings in the household of the child was defined as ≥1 sibling younger than 10 years living ≥3 days per week in the same house. A crowded living condition was defined as ≥ 5 inhabitants per household excluding the study subject and his/her siblings less than 10 years old.

Breastfeeding was evaluated combining the information collected at hospital discharge and that derived from the two follow up interviews, and was defined as feeding with maternal milk (exclusively or associated with formula), either from breast or bottle. Children were first classified as ‘never breastfed’ or ‘ever breastfed’, and children ‘ever breastfed’ were further classified according to having been fed exclusively with maternal milk or with both maternal milk and formula together.

Pollution exposure (i.e. residence in the proximity of intense traffic areas) and indoor humidity exposure (i.e. visible mold growth) were registered according to interviews. Exposure to epidemic season was defined as living for at least one month of their first three months of life from November to March.

Sample size was calculated by fixing a predefined precision of the estimate of the absolute risk of hospitalization and/or death for RSV-induced or non RSV-induced bronchiolitis during the first year of life. Assuming that the risk of hospitalization for bronchiolitis during the first year of life was about 7 %, a sample size of 2500 newborns could provide a 95 % confidence interval (95%CI) of 6.0 to 8.1 % that is largely consistent with a random error of less than 20 %.

The probability of bronchiolitis-related hospitalization was considered as a time-dependent variable, and a survival analysis was carried-out using Cox proportional hazard models to calculate the cumulative time-dependent risks. Relative risks were estimated as hazard ratios and 95 % confidence limits were derived from the standard errors of the parameter estimated by the model.

Crude hazard ratios were calculated for all the considered variables. Multivariable analyses were carried-out first on all the variables grouped within each set of pre-, peri-, and post-natal risk factors. All the variables significantly associated with the risk of hospitalization for bronchiolitis in these initial multivariable analyses entered into the final regression model.

Analyses were carried out using SPSS software package version 20.0 (IBM Corporation 2010; IBM SPSS Statistics for Windows).

## Results

In a three-year period, 2314 healthy newborns were enrolled. Out of those 2210 (95.5 %) of which 1150 male and 1060 female underwent a one year follow-up and were included in the analysis; 104 were lost to follow-up and were excluded. No infants died during the follow-up period.

Baseline demographic and clinical characteristics of the enrolled newborns and their parents by gestational age are reported in Tables 1, 2 and 3.

**Table 2:** Baseline demographic and clinical characteristic of the enrolled newborns and of their parents: perinatal/neonatal conditions

	wGA groups			
	33^+0d^-34^+6d^ (No. 737)	35^+0d^-37^+6d^ (No. 767)	≥38^+0d^ (No. 706)	Total (No. 2210)
Male gender	382 (51.8)	400 (52.2)	368 (52.1)	1150 (52.0)
Caesarean delivery	607 (82.4)	520 (67.8)	262 (37.1)	1389 (62,9)
Singleton delivery	419 (56.9)	552 (72)	702 (99.4)	1673 (75.7)
APGAR score at five minutes <8	42 (5.7)	22 (2.9)	7 (1.0)	71 (3.2)
Birth weight [g (mean, SD)]	2031 (380.9)	2605.1 (517.1)	3296.1 (423.6)	2635.1 (678.1)
Small for gestational age (SGA)	135 (18.3)	85 (11.1)	19 (2.7)	239 (10,8)
Resuscitation after birth	199 (27.0)	109 (14.2)	38 (5.4)	346 (15.7)
Neonatal hospitalization	715 (97.0)	430 (56.1)	137 (19.4)	1282 (58)
Respiratory diseases	296 (40.2)	121 (15.8)	23 (3.3)	440 (19.9)
Surfactant therapy	48 (6.5)	11 (1.4)	1 (0.1)	60 (2.7)
Antibiotics therapy	149 (20.2)	47 (6.1)	6 (0.8)	202 (9.1)

All data are reported as No. (%), unless otherwise specified; wGA = weeks of gestational age

**Table 3:** Baseline demographic and clinical characteristic of the enrolled newborns and of their parents: postnatal conditions

	wGA groups			
	33^+0d^-34^+6d^ (No. 737)	35^+0d^-37^+6d^ (No. 767)	≥38^+0d^ (No. 706)	Total (No. 2210)
Presence of siblings				
None	472 (64.0)	444 (57.9)	394 (55.8)	1310 (59.3)
≤ 10 year	223 (30.3)	289 (37.7)	280 (39.7)	792 (35.8)
> 10 year	42 (5.7)	34 (4.4)	32 (4.5)	108 (4.9)
Residential crowding	88 (11.9)	86 (11.2)	53 (7.5)	227 (10.3)
Lack of breastfeeding	209 (28.4)	193 (25.2)	80 (11.3)	482 (21.8)
Pollution exposure	77 (10.4)	102 (13.3)	87 (12.3)	266 (12.0)
Exposure to parental smoking	310 (42.1)	320 (41.7)	272 (38.5)	902 (40.8)
Indoor humidity	75 (10.2)	75 (9.8)	72 (10.2)	222 (10)
Exposure to epidemic season	437 (59.3)	474 (61.8)	412 (58.4)	1323 (59.9)
Hospitalization for bronchiolitis	54 (7.3)	41 (5.3)	25 (3.5)	120 (5.4)

All data are reported as No. (%); wGA = weeks of gestational age; yrs = years

Conception through assisted reproductive technology, multiple births, mothers with diseases potentially harmful for the pregnancy, or who were treated with corticosteroids, were more frequent in the group of babies born at 33-34 wGA. The babies in this group had a lower birth weight than newborns born at a longer gestational age and were more frequently SGA. In addition, they had more frequently neonatal respiratory diseases and/or received more frequently medical interventions (neonatal resuscitation in delivery room, hospitalization in neonatal department and/or in neonatal intensive care unit, surfactant and/or antibiotic therapy).

### Hospitalization for bronchiolitis during the first year of life

Out of the babies who underwent follow-up at 12 months, 120 (5.4 %) were admitted to hospital for bronchiolitis; 65/120 (54 %) hospitalizations occurred within the first 3 months of life and 90/120 (75 %) within the first 6 months of life. The majority of hospital admissions occurred during the RSV epidemic season and 95/120 (87.5 %) cases occurred in preterm infants, of whom 54/120 (45 %) were born at 33-34 wGA. The majority of the hospitalizations in this group occurred within the first 6 months (43/54, 79.6 %) of life, 31/54 (57 %) within the first 3 months of life.

Of the 120 hospitalized infants, 31/120 (26 %) were tested for RSV and 26/31 (83 %) were positive.

At univariate analysis (Table 4), the risk estimates (hazard ratios) for hospitalization for bronchiolitis increased 2 and 1.5 folds respectively for those born at 33-34 and 35-37 wGA compared to the newborns born at more than 37 wGA. Rates of bronchiolitis occurrence increased 1.6 folds for male newborns.

**Table 4:** Relative risks estimates (hazard ratios) according to prenatal, neonatal/perinatal and postnatal/environmental conditions for hospitalization

Exposures	Bronchiolitis hospitalization +/- exposed (%)		Multivariable HR^a^ (95%^b^)
	Events/unexposed	Events/exoposed [HR^c^, 95 % CI]	
Gender-male	44/1060 (4.2)	76/1150 (6.6) [1.6, 1.1-2.3]	1.6 (1.1-2.4)
Week of gestational age			
33-34 vs > 37	25/706 (3.5)	54/737 (7.3) [2.1, 1.3-3.4]	
35-37 vs > 37	25/706 (3.5)	41/767 (5.3) [1.5, 0.9-2.5]	
Prenatal			
Mother’s history of BPCO	117/2184 (5.4)	3/26 (11.5) [2.2, 0.7–6.9]	
Mother’s history of eczema	113/2123 (5.3)	7/87 (8.0) [1.6, 0.7–3.4]	
Father’s history of BPCO	115/2180 (5.3)	5/30 (16.7) [3.6, 1.5–8.9]	
Assisted reproductive technology	115/1959 (5.9)	5/251 (2.0) [0.3, 0.1–0.6]	
Treatment with corticosteroids	75/1645 (4.6)	45/565 (8.0) [1.4, 0.9–2.2]	1.6 (1.1–2.4)
Mother’s pregnancy pathologies	78/1623 (4.8)	42/587 (7.2) [1.4, 0.9–2.0]	
Prenatal smoke exposure	58/1358 (4.3)	62/852 (7.3) [1.8, 1.2–2.5]	1.6 (1.1–2.3)
Neonatal/Perinatal			
Delivery			
Singleton	23/537 (4.3)	97/1673 (5.8) [1.9, 1.2–3.0]	1.8 (1.1–2.9)
Resuscitation with O_2_	95/1911 (5.0)	25/299 (8.4) [1.4, 0.9–2.3)]	
Perinatal			
Neonatal hospitalization	83/1770 (4.7)	37/440 (8.4) [1.5, 1.0–2.2]	1.6 (1.0–2.5)
Surfactant therapy	109/2150 (5.1)	11/60 (18.3) [3.8, 1.6–5.8]	2.0 (1.1–3.8)
Postnatal (environmental)			
Having siblings < 10 years	40/1310 (3.1)	73/792 (9.2) [3.3, 2.2–4.8]	3.0 (2.0–4.5)
≥ 10 years	40/1310 (3.1)	7/108 (6.5) [2.2, 1.0–5.0]	1.9 (0.9–4.4)
Environmental (home) conditions			
Residential crowding	93/1983 (4.7)	27/227 (11.9) [2.6, 1.7–3.9]	2.4 (1.5–3.7)
Indoor humidity	101/1988 (5.1)	19/222 (8.6) [1.7, 1.0–2.8]	1.6 (1.0–2.6)
Heating system generating smoke	90/1742 (5.2)	30/468 (6.4) [1.2, 0.8–1.8]	
Residence in the proximity of intense vehicular traffic roads	100/1926 (5.2)	20/284 (7.0) [1.4, 0.9–2.3]	
Sibilings	63/1414 (4.5)	57/796 (7.2) [1.6, 1.1–2.3]	
Lack of breastfeeding	78/1728 (4.5)	42/482 (8.7) [1.8, 1.2-2.6]	1.8 (1.2-2.6)
Passive cigarette smoke exposure	112/2102 (5.3)	8/108 (7.4) [1.5, 0.7–3.1]	
Exposed to epidemic RSV season	31/887 (3.5)	89/1323 (6.7) [2.0, 1.3–3.0]	1.9 (1.3–2.9)

^a^Estimates from a model including all significant variables at gender and weeks of gestational age adjusted analysis

^b^95 % Confidence Interval

^c^Hazard ratios estimates adjusted for gender and weeks of gestational age

The prenatal, neonatal and postnatal conditions found to be significant at univariate analysis were included in the multivariate analysis (Table 4 and 5).

**Table 5:** Relative risks estimates (hazard ratios) of hospitalization for bronchiolitis in 33^+0d^-34^+6d^ wGA newborns

	Crude HR (95 % CI)	Adjusted HR (95 % CI)
Prenatal Conditions		
Assisted reproductive technology	0.3 (0.1-0.8)	
Prenatal smoke exposure	1.9 (1.1-3.3)	2.0 (1.2-3.5)
Neonatal Conditions		
Male gender	1.6 (1.1-2.3)	1.6 (1.0-2.7)
Singleton	1.7 (1.1-3.0)	
Apgar in the first 5 min <8	2.2 (1.1-4.9)	
Birth weight in g	1.0 (1.0-1.0)	
Surfactant therapy	3.8 (2.0-7.4)	3.1 (1.6-6.0)
Antibiotic therapy	2.0 (1.1-3.5)	
Postnatal Conditions		
Presence of siblings ≤10 year	3.8 (2.1-6.6)	3.2 (1.8-5.7)
Residential crowding	3.0 (1.7-5.5)	2.9 (1.6-5.4)
Indoor humidity	1.9 (1.1-3.8)	
Exposure to epidemic season	1.8 (1.0-3.0)	1.8 (1.0-3.3)

wGA = weeks of gestational age; HR = hazard risk; 95 %CI = 95 % confidence interval; yrs = years

At univariate analysis, the prenatal conditions that significantly increased the risk of hospitalization for bronchiolitis were: father history of COPD and prenatal exposure of the mother to active or passive smoking. Mother’s comorbid conditions (i.e. diabetes, hypertension) and treatment with corticosteroids showed an increased risk near statistical significance and were included in the multivariable analyses. Among the neonatal/perinatal risk factors, singleton delivery, respiratory diseases, surfactant therapy and lack of breastfeeding were associated to significantly higher risks of bronchiolitis admission at univariate analysis. Resuscitation with O_2_ at birth was associated with a 1.4 fold increased risk but reached only borderline statistical significance and was retained for subsequent analyses.

With regard to postnatal/environmental conditions, having siblings younger than 10 years, crowded living environment and being exposed to epidemic RSV season were associated with significantly higher rates of hospitalization. Residence in proximity of intense vehicular traffic determined a borderline statistically significant increase of risk for hospitalization for bronchiolitis and was retained for further multivariable analyses.

On multivariate analysis, a significant increase of the risk of hospitalization for bronchiolitis in the entire cohort was associated to male gender; prenatal treatment with corticosteroids; tobacco smoke exposure during pregnancy; singleton delivery; respiratory disorders and administration of surfactant at birth, lack of breastfeeding; siblings younger than 10 years; crowded living conditions and exposure to household humidity and exposure to epidemic season (Table 4).

Subgroup analysis on the group of preterm born at 33-34 wGA revealed that the significant risk factors partially differ from the results of the analysis performed on the entire group. At univariate analysis in this particular group, the significant risk factors for hospitalization for bronchiolitis are summarized in Table 5. At multivariate analysis only six factors maintained statistical significance: male gender; prenatal exposure to maternal smoking; neonatal surfactant therapy; having siblings younger than 10 years old, living in crowded conditions and exposure to epidemic RSV season (Table 5).

## Discussion

This study on infants born at 33 wGA or more provides an updated report on hospitalizations for bronchiolitis within an Italian network of thirty Neonatology and Pediatric Units in a large longitudinal birth cohort. When analyzing the entire cohort, we found that about 5 % of the newborns were admitted to hospital for bronchiolitis during their first year of life as well as preterm infants born at 33-34 wGA had a higher risk of hospitalization compared to those born at ≥35wGA. The criteria for hospitalization in Italy were previously proposed by several pediatric scientific society and have been summarized in an inter-society consensus document in 2014 [21]. The indications to hospitalization include clinical evaluation and analysis of pre-existing risk factors such as prematurity, congenital heart diseases or chronic diseases. Given the presence of shared national guidelines, the decision to hospitalize an infant to be derived from the analysis of all the individual criteria. Male gender, prenatal conditions (treatment with corticosteroids and exposure to tobacco smoking), neonatal conditions (singleton delivery, respiratory diseases, surfactant therapy) and environmental/postnatal conditions (lack of breastfeeding, having siblings <10 years old, crowded and unhealthy living conditions and being exposed to epidemic RSV season) were associated with a significant increase of hospitalization rate for bronchiolitis in the entire birth cohort.

The same analysis repeated in the group of infants born at 33-34 wGA proved that prenatal smoke exposure, male gender, surfactant therapy, presence of siblings < 10 years, crowded living conditions and exposure to epidemic RSV season are statistically significant risk factors for hospitalization for bronchiolitis.

The results of our analyses were adjusted for wGA, when referring to the whole cohort, or restricted to 33-34 wGA infants. According to this, our results point out that, beyond to postnatal exposures (independent from wGA), pre- and peri-natal risk factors are associated to conditions of clinical severity differently prevalent in premature or non-premature infants, so that prematurity “per se” is not to be considered a predictor.

Although severe diseases are more common among children with prematurity, CLD, CHD and other pathologic conditions, more than half of babies hospitalized for RSV infections during the first year of life [1] and 80 % of RSV-related deaths occur among children who do not have an underlying clinical high-risk condition [4, 22]. This suggests that environmental or other conditions enforce the risk condition due to prematurity and pre-existing diseases, determining an additional increase of the risk [23, 24, 10].

Our results only partially agree with other studies. These disagreements seem intrinsic to the nature of cohort studies, moreover when carried out in different settings and with different methodologies. We can hypothesize that study procedures, different populations, data collection, variable definitions and follow-up duration may have played a role in determining partially different results.

Male sex is known to be a risk factor for severe RSV bronchiolitis [23, 14] with a risk ratio of boys to girls being 1.425:1 [23].

It is also well known that smoking in pregnancy is associated with reduced lung function of the newborn [25–27]. In a large case–control study from Denmark [28] that was designed to evaluate risk factors for RSV hospitalization in infants younger than 2 years, prenatal exposure to smoking was one of the significant factors on multivariate analysis (odds ratio: 1.56; 95 % CI: 1.32–1.98).

In our study, prenatal corticosteroid administration and singleton birth were observed to increase the risk for hospitalization for bronchiolitis. These data are discordant with previous studies and require further confirmation [29, 30].

A perinatal/neonatal history of respiratory diseases was observed to be a risk factor for hospitalization, since any condition impairing the early postnatal development of the lung predisposes infants to an increased risk of severe bronchiolitis.

Different papers reported conflicting results about the protective role of breastfeeding on lower respiratory infections. A preliminary analysis conducted on this birth cohort study reported a clear significant reduction of the risk of hospitalization for bronchiolitis associated with breastfeeding (exclusive or in addition to formula milk) [31], confirmed by the analysis performed in the present paper on the entire cohort. The studies by Holberg et al. [32] and Bulkow et al. [33] also described a decreased risk of RSV hospitalization in breastfeeding infants.

Both crowded living conditions and presence of siblings appear to be important risk factors for more severe acute respiratory infections. Reasons include the increased likelihood of exposure to the virus circulation and subsequently the increased risk for infection. In the Canadian PICNIC study [14], the presence of preschool-aged siblings was significantly and independently associated with an increased risk for RSV related hospitalization, and a weaker association was found with the presence of school-aged siblings. Crowded living conditions, defined as 5 or more people living in one household, was also demonstrated to be a significant risk factor for RSV related hospitalization. In some contrast, the Spanish FLIP study [13] revealed that only school-aged siblings and the presence of more than four additional residents and visitors at home were risk factors significantly associated with RSV related hospitalization. In the FLIP-2 study [12], the effect of school-aged siblings was confirmed, however crowded living conditions, identified by the same definition, was not. The Munich RSV Study Group [7], found that siblings at day care attendance significantly augmented the risk for RSV related hospitalization (OR: 3.9; 95 % CI: 1.9-8.3).

Young age at the beginning of the RSV season is a risk factor for both the development of LRTI as well as hospitalization due to RSV infection. A review of studies of RSV hospitalization rates [23] revealed that approximately 10 % to 28 % of infants hospitalized with RSV are aged below 6 weeks, 49 % to 70 % below 6 months, and 66 % to 100 % below one year.

Moreover, a recent study [34] analyzing hospitalizations for acute respiratory infections in infants < 24 months reported that the hospitalization rate was higher during the first three months of life and then consistently declined.

The greatest risk factor for hospitalization due to RSV infection appears to be the first few months of life when coincided with the first half of the RSV season. This data was also confirmed by two Spanish studies from the IRIS Group [12, 13] showing that infants with a chronological age below 10 weeks at the onset of the RSV season were at higher risk for RSV related hospitalization. In addition, the majority of hospitalization in our cohort occurred during the first 6 months of life and this result was confirmed focusing the attention in the subgroup of infants born at 33-34 wGA.

This study has some limitations. Firstly, microbiological data to confirm the presence of RSV was available only in 26 % of the cases of hospitalized infants, nevertheless with a percentage of RSV positivity higher than 80 %. Previous Italian data [19] are similar to our data (near 15 % of observed clinical bronchiolitis in hospitalized infants were negative when was tested for RSV).

Moreover, a large Italian study conducted on infants < 2 years hospitalized for LRTI [15] reported that 40.6 % of tested infants were RSV positive and the majority of RSV bronchiolitis occurred in infants ≤ 3 months.

These data suggest that the rate of etiologic misclassification can be considered low, also because of the age of our study subjects being younger than one year, at which 72-84 % of cases of bronchiolitis are RSV positive [35].

In addition, a previous study in the UK estimated that around 75 % of unspecified bronchiolitis admissions were RSV related [36]. Globally, it is estimated that only between 4 % and 28 % of children admitted with bronchiolitis are tested for RSV [3].

Considering the statistical analysis, when simultaneously analyzing several covariates, issues about overfitting, interaction and correlation may arise. Different not resolutive approaches and techniques, with relative pros and cons, may be adopted to address these issues. We think the potential overfitting was adequately controlled in our analyses by applying the multi-step selection of the variables that led to a reduced number of predictors entering the final models. Interaction is a complex issue that can be handled with different statistical techniques, none of them exhaustive, among which one of the mostly adopted is the building of mutually exclusive levels in new combined variables, as performed by Escobar et al. [37]. However, in our study no statistically significant interaction was found in the final set of predictors, so we think that the parsimonious models we proposed were appropriate for these analyses.

The strength of this study is that it is the first study to focus on a large longitudinal Italian birth cohort of newborns in order to identify rates and risk factors for hospitalization for bronchiolitis.

This could allow to improve the indications of prophylaxis with palivizumab in infants born at 33 weeks or more (particularly those born at 33-35wGA), reducing both the hospitalizations for acute bronchiolitis and its possible long-term sequelae.

Besides developing particularly serious RSV infections in the first year of life, preterm infants, even those without CLD, are at higher risk for developing recurrent wheezing, asthma [38] and allergic sensitization [39–42] and have persistent abnormal lung function [43]. Moreover in a recent double-blind, placebo-controlled MAKI trial on healthy preterm infants born at a gestational age of 33 to 35 weeks, Blanken et al. [44] demonstrated that palivizumab treatment significantly reduces wheezing days during the first year of life, even after the end of treatment.

## Conclusions

The analysis of local epidemiological data and risk factors involved in RSV-related hospitalization is mandatory in order to better plan preventive strategy and to develop updated national guidelines tailored for pediatric high-risk populations. Epidemiological data and a number of underlying risk factors that significantly increase the risk of severe bronchiolitis and subsequent hospitalization rate in this group of infants have been identified in our national birth cohort study, confirming that an infant’s individual characteristics and exposure to environmental factors play an important role in determining the risk of severe infection and hospitalization, independently from preterm birth.

The analysis of the weight of each risk factor could allow to define with greater accuracy the risk for bronchiolitis hospitalization for any infant during the first year of life and to take into account the prophylaxis with palivizumab through the determination of “tailored” indications.

## Abbreviations

CHD: Congenital heart disease
CLD: Chronic lung disease
COPD: Chronic obstructive pulmonary disease
GA: Gestational age
LRTI: Lower respiratory tract infections
RSV: Respiratory syncytial virus
SGA: Small for gestational age
wGA: Weeks of gestational age
